# The Role of a Medical Journal

**DOI:** 10.1016/j.shj.2022.100079

**Published:** 2022-08-29

**Authors:** Anthony N. DeMaria

**Affiliations:** Judy and Jack White Chair in Cardiology, Sulpizio Cardiovascular Center, University of California, San Diego, La Jolla, California, USA

I recently participated in the Dialogues program of the *European Heart Journal* initiated by its Editor, Filippo Crea. The format is a relatively open-ended discussion between an individual and the editors of the journal. The conversation touched upon my personal background and career evolution, the origination, development and future of cardiac ultrasound, and thoughts about various editorships that I had held. Significantly, during the discussion, I was asked my opinion about the proper role of medical journals. That stimulated me to rethink the answer and ultimately to write this Editor’s Page.

The origin of the modern peer review medical journal is usually credited to Henry Oldenburg, who is described as a theologian and natural philosopher. He was the founding editor of the Philosophical Transactions of the Royal Society (of London). Prior to this publication, the transmission of information largely consisted of correspondence between individuals. The Philosophical Transactions provided a central home for the correspondence. Of greater significance, Oldenburg was the first to send out communication to other authorities to determine the worthiness for publication. The Philosophical Transactions represented the best new information in the field that had been judged by peers to be accurate and worthy of being read. Thus was the process of peer review established.

Opinions differ regarding the goals of a medical journal, and therefore, the potential contents can vary greatly. Journals may serve to provide novel observations or knowledge or to consolidate and review existing knowledge. They can offer opinion or news about a professional society or society in general. They can supply information regarding the logistics or economics of providing care. In the current environment of internet communications, they can provide videos of real-time imaging and even demonstrate the performance of procedures. Not surprisingly, a review of current medical journals reveals a broad spectrum of content.

Journals are the traditional venue for communicating new research findings. The process invariably involves peer review whereby the data are scrutinized by experts to ensure accuracy, novelty, and relevance. The journals may vary in focus, such as basic research vs. clinical investigation. To varying degrees, they may offer editorial comments regarding the data presented. They may differ regarding the detail in which methodology is described. Nevertheless, nearly all journals exist to publish the results of new research. As an adjunct, most journals will attempt to educate by providing scholarly reviews of important topics. The best of these reviews will not only present the existing data but also organize and synthesize the information to draw consensus conclusions and/or identify unresolved issues. The epitome of a scholarly review is a guideline or a consensus document authored by a group of authorities or stakeholders designed to guide practice. Such manuscripts are invariably the most read and cited. While most journals contain some review material, a few journals are comprised only of review articles. Original research findings and review articles represent the core content of most journals.

A plethora of material complements the research and review content. Case reports are very prevalent as are sections devoted to images or procedural vignettes. While case reports and vignettes occasionally present original material, more often they describe material that is rare but has been previously reported. Articles regarding health care delivery and/or quality are often included in content. If the journal is an organ of a medical society, it will often include information relevant to its activities. Recently journals have become more creative in their use of online communications. Detailed how-to presentations are sometimes provided in a video format for new and even well-established procedures such as lumbar puncture. An overview of the material in any issue may be presented in either video or audio format by the editor or their designee. The Dialogues program of the EHJ is an example of one of the more innovative initiatives of a journal to provide information to its readers, or in this case, viewers. Thus, journals that started as a venue for the presentation of original peer-reviewed papers have morphed into multimedia publications with a wide range of roles.

In my opinion the primary, most important and critical role of a medical journal is to present the findings of original peer-reviewed research. I applaud utilizing the opportunity that journals provide to deliver education in a variety of formats. Review papers in medical journals should be the highest quality available since they have passed peer review and presumably have been screened to be free of bias. Similarly, it is crucial to have the involvement of the profession in issues of health care delivery and quality, so this content is also valuable. There is little question that exploiting the advantages inherent in online presentation enables journals to both educate and, to some degree, entertain. So there are many valuable roles that journals can fill. However, the opportunities for medical education are ubiquitous. Review articles are readily available from a variety of venues, as is material in other formats including the internet and meetings. Conversely, only medical journals can provide the results of original research that has undergone peer review by experts. It is true that peer review may be imperfect, and it is susceptible to the subjectivity of reviewers and editors. But like democracy, it is the best system available. When published in a medical journal, one can have the maximal confidence possible of the validity of a paper. Of equal importance, all research reports must be viewed with some caution until the data pass the crucible of peer review by experts and are judged deserving of publication in a journal. So as a medical editor, I spend the vast majority of my time and effort evaluating original research and spend less time on educational content.

## Funding

The author has no funding to report.

## Disclosure statement

The author reports no conflict of interest.

